# Accuracy, precision, and efficiency of manual planimetry versus point counting for sectional area estimation in oral morphometry: a comparison using a simulated two-dimensional reference model

**DOI:** 10.3389/froh.2026.1897801

**Published:** 2026-09-07

**Authors:** Chengqi Zhang, Yupiao Chen, Dongyun Wang, Yuelian Liu, Yunlin Zhang, Xingnan Lin

**Affiliations:** 1 School of Stomatology, Zhejiang Chinese Medical University, Hangzhou, Zhejiang, China; 2 Department of Stomatology, Peking University Shenzhen Hospital, Shenzhen, Guangdong, China; 3 Academic Centre for Dentistry Amsterdam (ACTA), University of Amsterdam and Vrije Universiteit Amsterdam, Amsterdam, Netherlands; 4 Liangmu Dental Clinic, Nanjing, Jiangsu, China; 5 Department of Stomatology, Taikang Xianlin Drum Tower Hospital, Affiliated Hospital of Medical School, Nanjing University, Nanjing, Jiangsu, China

**Keywords:** cavalieri principle, manual planimetry, oral morphometry, point counting, sectional area estimation, stereology

## Abstract

**Background:**

Reliable sectional area measurement is essential for quantitative analysis in dental imaging, histomorphometry, and guided surgery. Although manual planimetry and point counting are widely used, their comparative measurement performance at the isolated two-dimensional level has seldom been evaluated against a predefined nominal reference.

**Materials and methods:**

A simulated two-dimensional nominal reference model was created using 30 artificial sections with mathematically predefined true areas. Two observers independently measured the area of each section with both methods; all measurements were repeated after a two-week washout period. For each method, repeatability, absolute bias relative to the true area, and time required per section were evaluated. Bland–Altman analysis was used to characterize the structural patterns of measurement error.

**Results:**

Both methods showed excellent repeatability. However, point counting yielded a significantly smaller average bias and required less time per section than manual planimetry. Bland–Altman analysis revealed distinct error profiles: manual planimetry was characterized by systematic underestimation that increased with section size and geometric complexity, whereas point counting maintained consistent accuracy across the entire range of section areas.

**Conclusions:**

Under the controlled two-dimensional workflows tested, point counting showed smaller bias and greater efficiency than manual planimetry. However, because planimetry was performed on scanned images whereas point counting was performed directly on physical sections, these findings should be interpreted as a comparison of the tested implementations rather than evidence of intrinsic methodological superiority. Manual planimetry, although highly repeatable, was susceptible to systematic underestimation, particularly when delineating complex and fragmented contours. These findings may inform methodological selection in morphometric studies, but their relevance to automated segmentation evaluation requires further validation in clinical or histological datasets.

## 1 Introduction

Quantitative assessment of anatomical structures, particularly lesion extent and tissue dimensions, is essential in oral medicine, oral pathology, and oral surgery (1, 2). In histomorphometric analysis and guided surgical planning alike, objective 2D sectional area measurement constitutes a critical diagnostic metric and a prerequisite for reliable volumetric reconstruction. The reliability of these sectional measurements is therefore of paramount methodological importance across a broad range of dental applications.

For three-dimensional quantification, the Cavalieri principle offers a well-established stereological framework in which volume is estimated by multiplying the sum of sectional areas by a constant intersection interval (3, 4). The validity of the resulting volumetric estimate depends directly on the accuracy of the underlying 2D sectional area measurements (3, 5, 6).

Manual planimetry and point counting represent two of the most commonly employed techniques for area estimation. Manual planimetry requires the operator to trace the entire perimeter of the region of interest on each section continuously. Point counting, by contrast, estimates area by overlaying a systematic grid of test points and counting the number of “hits,” each weighted by the area per point. Although the two methods perform comparably in CT and MRI volumetry, they differ markedly in workflow and time demand (7, 8).

Despite their widespread use, the comparative performance of these techniques at the fundamental 2D level has rarely been validated against a predefined nominal reference (9, 10). Previous studies have largely relied on method-to-method agreement or external 3D volumetric standards such as fluid displacement (6, 11). While informative, volume-level discrepancies in such investigations may stem not only from errors inherent in the area measurement method itself but also from confounding z-axis sampling factors, including section thickness and inter-section spacing (5). Volume-level validation alone, therefore, cannot establish whether one area estimation technique is intrinsically more accurate than the other.

To address this gap, we evaluated manual planimetry and point counting at the isolated 2D level, deliberately eliminating z-axis sampling interference. Unlike clinical images, which are often degraded by noise and ambiguous boundaries, the present study employed a high-contrast physical model with explicitly defined borders. This design removed image-quality artifacts and isolated the intrinsic mathematical and operational errors of each measurement technique. However, we acknowledge that the two methods were performed on different media: planimetry was conducted on scanned 600 dpi images using Qwin software, whereas point counting was performed directly on the physical sections with a transparent overlay grid. This methodological difference means that the observed bias in planimetry may partially reflect the complete scanned-image tracing workflow, including boundary interpretation, thresholding-related edge effects, calibration procedures, and manual tracing behavior, rather than tracing error alone. Using a controlled series of artificial sections with mathematically predefined true areas as a simulated 2D ground-truth reference, we systematically compared the accuracy, precision, and efficiency of the two methods. While our findings indicate that point counting achieved smaller bias under the conditions tested, we refrain from claiming that it is intrinsically more accurate at the fundamental mathematical level; rather, the comparison reflects the performance of these specific implementations, which differed in both medium and operational workflow. The findings clarify the fundamental measurement performance that underlies Cavalieri-based morphometry and oral morphometric workflows.

## 2 Materials and methods

### 2.1 Development of a simulated two-dimensional nominal reference model

A physical model comprising 30 artificial sections was constructed to serve as a reliable reference standard. Standardized white graph paper (5 × 5 mm grid) provided the background, while sheets of red paper of a precisely known area (89 × 89 mm²) were used as the benchmark unit. This design emulated the irregular, fragmented, and geometrically complex configurations typical of biological and histomorphometric sections.

The benchmark red sheets were first assigned into known fractional portions to define the nominal area allocated to each section. These portions were then cut into irregular, non-rectangular polygonal fragments and assembled on the graph-paper background in arbitrary positions and orientations. Adjacent fragments were kept visibly separated, with an approximately 2–3 mm inter-fragment gap where possible, to avoid overlap or contact; any fragments that inadvertently overlapped were discarded and replaced. The graph paper served as a scale reference and background support, but fragment edges were not constrained to align with the grid lines. As a bookkeeping consistency check, the intended nominal area of each section was calculated by summing the known fractional areas of its constituent pieces and comparing this value with the predefined formula A(i) = (1 + i/8) × 89 × 89 mm². This check confirmed internal consistency of the intended design, but it was not an independent physical measurement of the assembled section area. The area of the first section was set to 1 + 1/8 benchmark units; each subsequent section received an additional 1/8 unit, up to the 30th section. Thus, the nominal area of interest (AOI) for section i was defined as A(i) = (1 + i/8) × 89 × 89 mm² (i = 1, 2, .., 30). As the nominal area increased, the sections became progressively more fragmented and possessed longer total boundary perimeters, thereby creating targets of increasing geometric complexity.

To minimize manufacturing errors, 250 g/m² red cardstock was selected to resist curling and moisture-induced deformation. All cutting was performed with a precision rotary cutter against a metal ruler on a self-healing mat; cut edges were inspected under 5× magnification to exclude fraying or irregularities. Prior to assembly, sheets were conditioned for 24 h under controlled laboratory conditions (20–22 °C, 45%–55% relative humidity) to stabilize dimensions. Fragments were adhered to the background graph paper using a minimal amount of acid-free, repositionable adhesive applied only at the periphery, with care taken to prevent overlap, gaps, or wrinkles. The assembled sections were pressed overnight under a flat acrylic plate (approximately 2 kg) to ensure complete planarity. These procedures were used to minimize construction-related deformation, overlap, and wrinkling. However, no substrate-independent physical determination of fragment area, such as gravimetric measurement based on paper mass and areal density, was performed. Therefore, the predefined areas should be interpreted as nominal geometric reference values constructed under controlled conditions rather than independently measured physical ground-truth areas.

**Figure 1:**
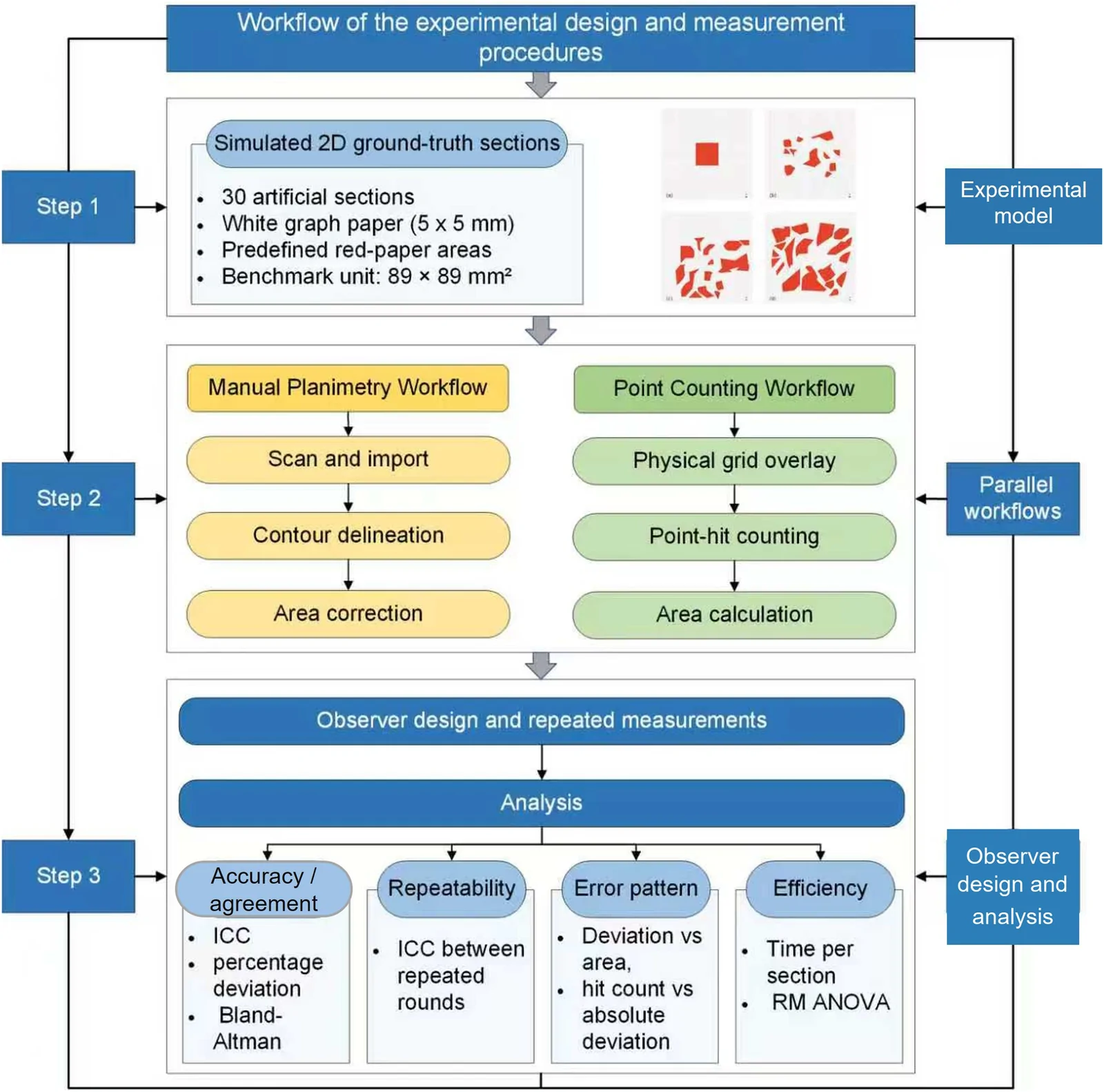
Simplified experimental workflow. Artificial sections with predefined true areas served as a simulated two-dimensional nominal reference. The inset shows representative sections: **(A)** benchmark sheet, **(B)** sample 1, **(C)** sample 15, and **(D)** sample 30. Sections were evaluated by manual planimetry and point counting, followed by repeated measurements and analyses.

A computer-generated schematic of the benchmark sheet and selected sections is shown in the upper-right corner of Figure 1, rather than photographs of the physical samples.

### 2.2 Manual planimetry protocol

The 30 physical sections were digitized at 600 dpi with a flatbed scanner (Ricoh Aficio MP C4502, Japan) and saved as TIFF files. Area measurement was carried out in the Qwin image analysis software (Leica Microsystems). The operator carefully traced the outermost boundary of every discrete red fragment using the polygon selection tool. The total AOI per section was then obtained automatically by summing all selected sub-areas. To correct for any scanning-induced dimensional distortion, image magnification was calibrated against the 5 × 5 mm grid of the background paper before measurement. For each section, the operator started a digital stopwatch immediately before initiating the polygon tracing and stopped it as soon as the final vertex was closed and the software area readout was recorded. This per-section interval excluded preparatory steps such as image import, magnification calibration, and file saving, so that the recorded time reflected only the active manual delineation process. Despite this calibration step, residual scanning artifacts, including minor thresholding variations at fragment boundaries and subpixel geometric distortions, may have contributed to measurement error independently of the tracing process itself.

### 2.3 Point counting protocol

To avoid digital artifacts, point counting was performed directly on the physical sections. A transparent overlay bearing a systematic point grid (1.25 cm spacing) was placed over each section at a 1:1 scale. Points falling on the AOI were counted; a point was considered a “hit” if it lay completely within a fragment or if the upper-right quadrant of the cross-mark intersected the fragment boundary (12). The area was then calculated as A = *n* × d², where *n* is the hit count and d is the grid spacing. The grid spacing was chosen to guarantee at least 50 hits even on the smallest section; across all observer–round measurements, the observed hit counts ranged from 51 to 241 per section.

Timing followed the same stopwatch protocol: the observer started the timer immediately before placing the transparent grid over the section and stopped it immediately after the last point had been counted and the hit tally recorded. Grid placement and removal were therefore included in the measured interval, as they constitute inseparable on-section operational steps, whereas pre-session grid preparation and between-section pauses were excluded.

Precision of the point counting estimates was assessed using the Gundersen–Jensen coefficient of error (CE), the standard precision metric for systematic random sampling in stereology (13). Across all 120 observer–round measurements, the per-measurement CE was approximated as 1/√P, where *P* is the recorded hit count; values ranged from 6.44% (241 hits) to 14.00% (51 hits), with a mean of 8.74%. At the study level, the Gundersen–Jensen CE was calculated separately for each observer–round series of 30 sections according to the smoothness class m = 1 estimator:

CE=√(TotalVar)/ΣP_i

where $TotalVar\;=\;s^{2}+VAR\_SRS,$

$s^{2}=0.0724\times (b/\surd a)\times \surd (n\times \Sigma P\_i)$ [noise variance],

$VAR\_SRS=[3(A-s^{2})-4B\;+\;C]/240$ [systematic sampling variance],

with $A=\Sigma P\_i^{2}$, B=Σ(P_i×P_{i+1}), C=Σ(P_i×P_{i+2}), *n* = 30 sections, and a shape factor (b/√a) of 5.5 for irregular fragmented profiles. The study-level CE values were 0.44%, 0.46%, 0.43%, and 0.44% for Observer 1 round 1, Observer 1 round 2, Observer 2 round 1, and Observer 2 round 2, respectively; all values (range, 0.43%–0.46%) were well below the conventional 5% threshold for acceptable stereological precision.

The overall study design, the two measurement workflows, and the subsequent observer and statistical procedures are summarized in Figure 1.

### 2.4 Intra- and interobserver variability

Two observers, both experienced in using Qwin and the point-counting method, independently measured the AOI on all 30 sections. Before each round, the sections were relabeled with new codes and presented in a freshly randomized order. Thus, each of the four assessment sessions (observer 1, rounds 1 and 2; observer 2, rounds 1 and 2) was conducted on a different randomized sequence. Both observers were blinded to the true area of each section and to the size progression used during construction. Each observer performed both methods twice, with a two-week washout between repeated measurements (14). Reproducibility was primarily assessed with intraclass correlation coefficients (ICC), with coefficients of variation (CV) reported as supplementary descriptive statistics.

### 2.5 Statistical analysis

**Figure 2:**
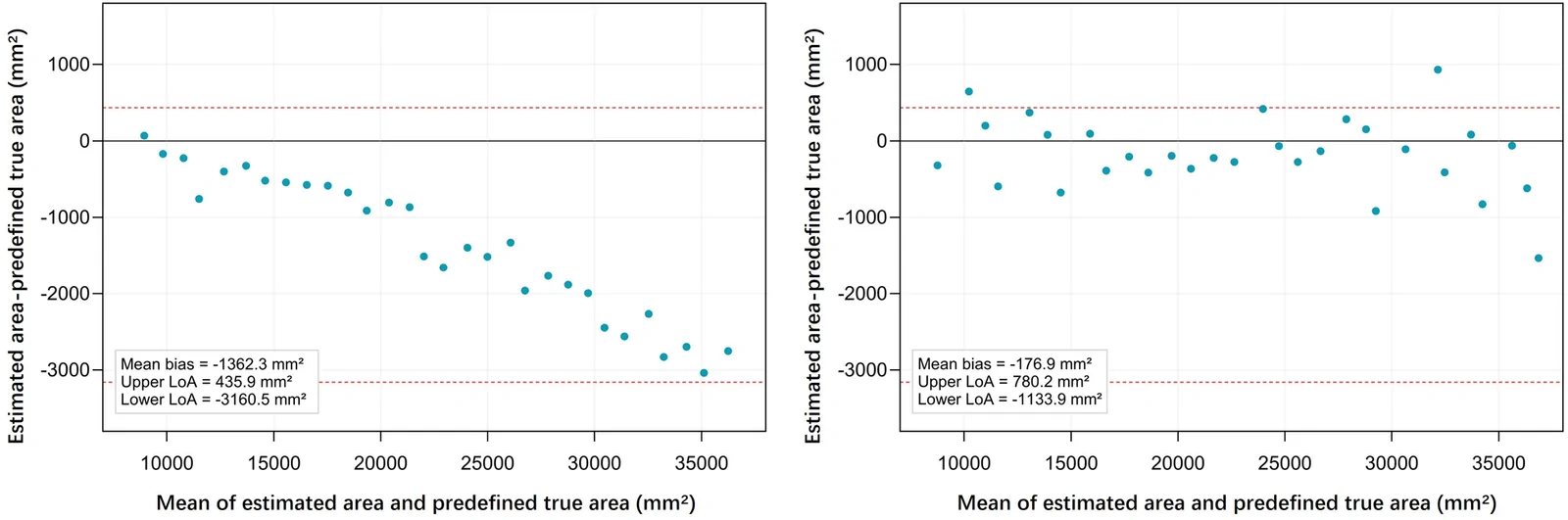
Time required per section for area estimation by point counting and manual planimetry. Each point represents the mean of two repeated timing measurements for one section (*n* = 30 section-level values per observer–method combination). Bars represent the means of these 30 section-level values, and error bars indicate standard deviations. Brackets indicate comparisons between point counting and manual planimetry within each observer (Šídák-adjusted *P* < 0.0001 for both comparisons).

Statistical analyses addressed three domains: accuracy, precision, and efficiency. Intraclass correlation coefficients (two-way mixed model, absolute agreement) were computed to evaluate agreement with the true area (estimated vs. nominal reference area) and within-method precision (test-retest reliability). Measurement bias was expressed as percentage deviation: [(Estimated Area−True Area)/True Area] × 100, with negative values indicating underestimation. Bland–Altman plots were generated to visualize mean bias and 95% limits of agreement. A two-way repeated-measures ANOVA was used to compare the time required per section across methods and observers. Time was recorded separately for each section, observer, method, and round, yielding 240 raw timing observations in total (30 sections×2 observers×2 methods×2 rounds), corresponding to 120 observations per method. For each section and observer–method combination, the two round-specific times were averaged, yielding 30 section-level mean times per combination. These section-level values were used in the two-way repeated-measures ANOVA, with method and observer treated as within-section factors, and to calculate the descriptive means and standard deviations shown in Figure 2. The complete raw timing dataset is provided in Supplementary Table S1.

## 3 Results

The experimental model yielded a wide range of true areas, from 8,911.0 mm² to 37,625.0 mm² (mean = 23,268.0 ± 8,716.5 mm²).

### 3.1 Accuracy

Both methods showed excellent absolute agreement with the true areas, with all ICC values exceeding 0.97 across observers and rounds (Table 1). Point counting produced marginally higher ICCs than manual planimetry.

**Table 1:** ICCs for accuracy and within-method reproducibility of point counting and manual planimetry.

Observer	Round	Point counting (accuracy)	Point counting (E1 vs. E2)	Planimetry (accuracy)	Within planimetry (E1 vs. E2)
Observer 1	E1	0.995	0.993	0.978	0.998
	E2	0.991		0.974	
	Average	0.994		0.976	
Observer 2	E1	0.993	0.988	0.983	0.999
	E2	0.993		0.984	
	Average	0.996		0.984	

Accuracy ICCs indicate agreement between estimated area and the predefined true area. E1, first measurement round; E2, second measurement round; Average, mean of E1 and E2. Within-method reproducibility ICCs indicate agreement between E1 and E2 for each observer within the same method.

Analysis of percentage deviation revealed the magnitude and direction of bias (Table 2). Manual planimetry consistently underestimated the true area. For Observer 1, the mean deviations were −6.05% and −7.77% in the two rounds, with narrow standard deviations, indicating high consistency but a clear systematic bias. Point counting estimates, in contrast, were distributed much closer to the true values. Although their standard deviations were slightly larger, suggesting greater random variation, the mean bias was markedly smaller. Paired t-tests confirmed that the deviation from the true area was significantly larger for manual planimetry than for point counting in all comparisons (*P* < 0.001). Because the 30 sections represented a graded, non-independent series derived from the same benchmark sheet with progressively increasing areas, the four paired comparisons (Observer 1 round 1, Observer 1 round 2, Observer 2 round 1, Observer 2 round 2) are not statistically independent. No formal multiple-comparison adjustment (e.g., Bonferroni or Šídák correction) was applied; instead, the consistency of the effect across all four comparisons is emphasized as supporting evidence rather than as four independent confirmations of significance.

**Table 2:** Percentage deviation of the estimated area from the predefined true area.

	Point counting				Planimetry			
Statistic	Observer 1(E1)	Observer 1(E2)	Observer 2(E1)	Observer 2(E2)	Observer 1(E1)	Observer 1(E2)	Observer 2(E1)	Observer 2(E2)
Mean	−2.16	−4.61	3.06	1.10	−6.05	−7.77	−3.59	−3.15
SD	3.58	3.16	3.86	5.08	1.94	1.31	3.36	3.70
Max	4.73	2.08	13.62	18.6	−1.37	−4.65	4.25	4.92
Min	−10.57	−11.63	−4.26	−7.12	−8.94	−10.39	−8.46	−8.27

Percentage deviation was calculated as (estimated−true)/true×100. Negative values indicate underestimation and positive values indicate overestimation. E1, first measurement round; E2, second measurement round. SD, standard deviation; Min, minimum; Max, maximum.

**Figure 3:**
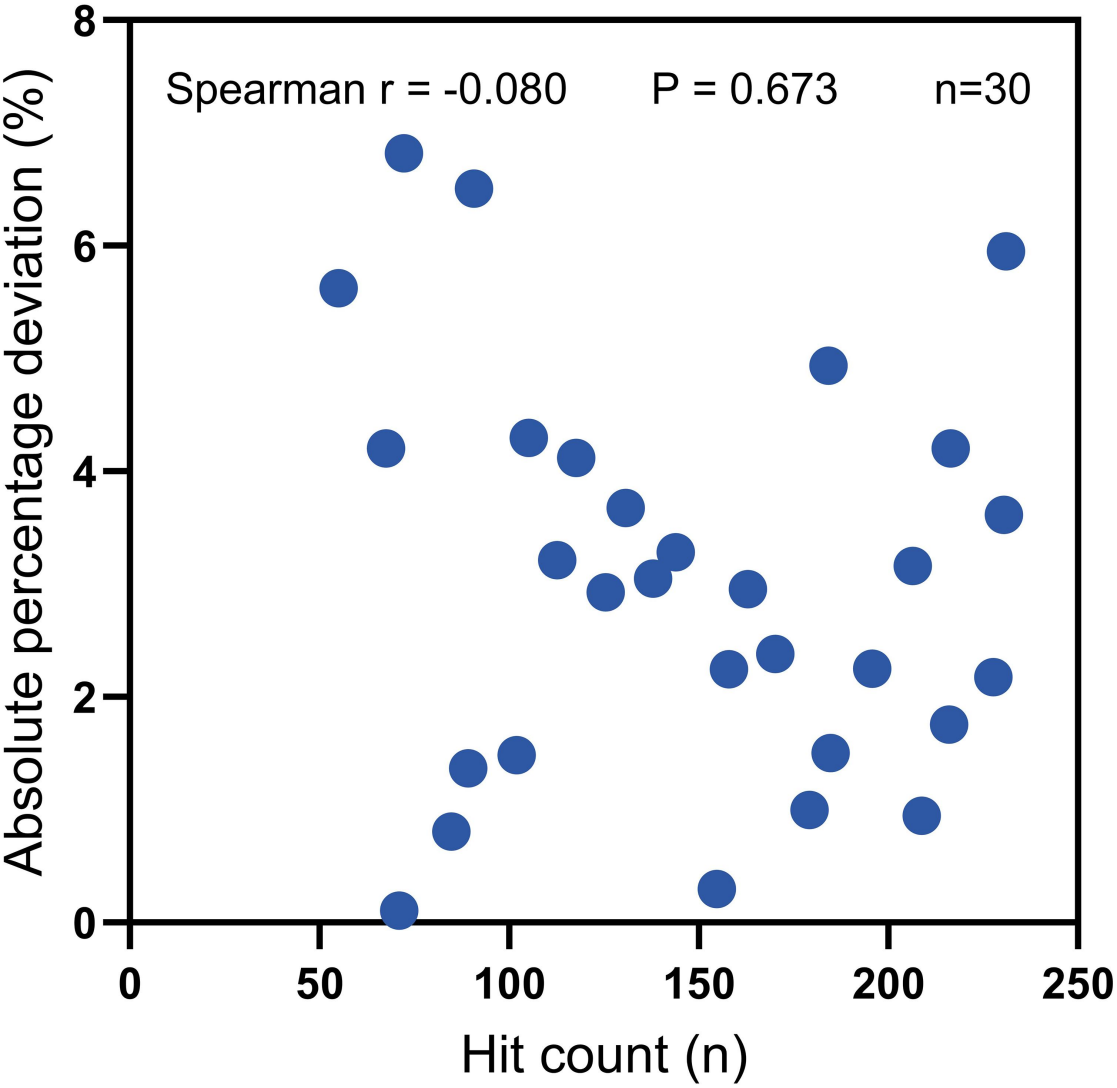
Bland–Altman analysis of agreement with the predefined true area. **(A)** Manual planimetry. **(B)** Point counting. Each point represents one section. The *x*-axis shows the mean of the estimated and predefined true areas, and the *y*-axis shows the difference between them. Dashed lines indicate the mean bias and 95% limits of agreement.

Bland–Altman analysis further illustrated this structural difference (Figure 3). Point counting had a mean bias of −176.9 mm² and relatively narrow 95% limits of agreement (−1,133.9–780.2 mm²). Manual planimetry, by comparison, showed a large negative bias (−1,362.3 mm²) and wider limits of agreement (−3,160.5–435.9 mm²). Importantly, the planimetry plot displayed a clear proportional error: the degree of underestimation increased as the true section area became larger and more geometrically complex.

### 3.2 Association between true area and percentage deviation

**Figure 4:**
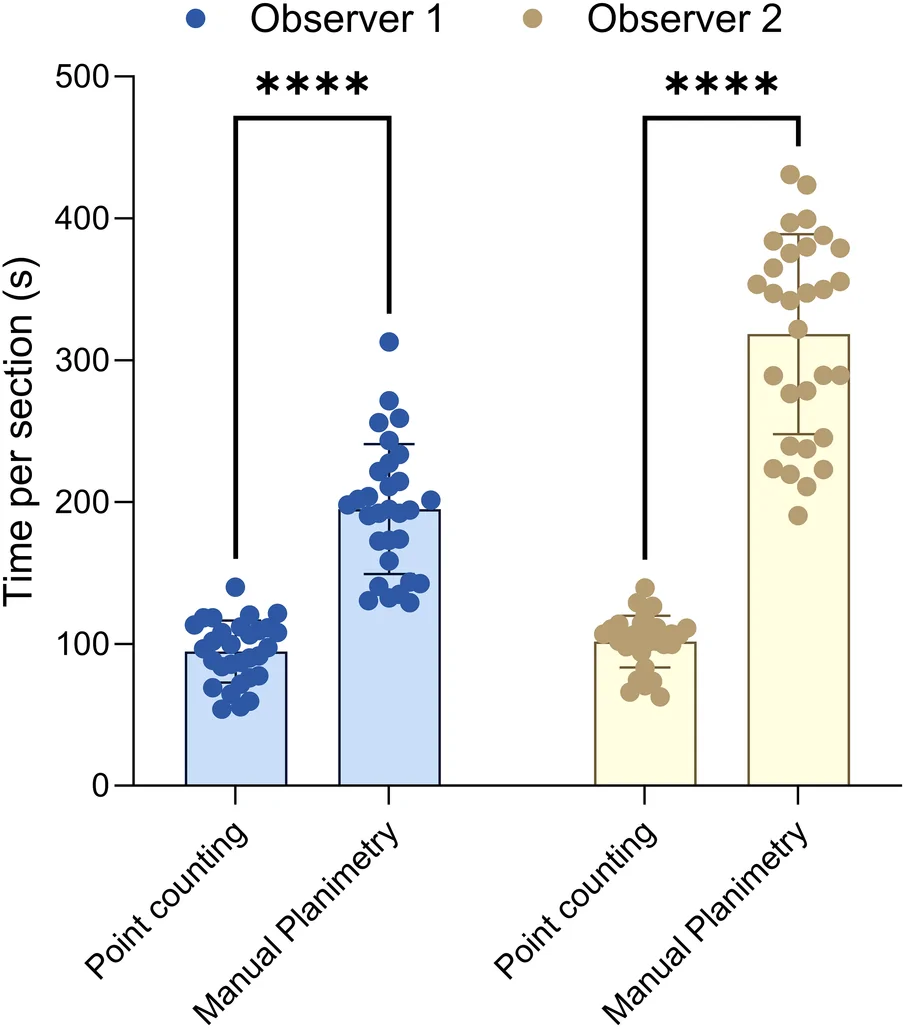
Association between hit count and absolute percentage deviation in point counting. Each point represents one section, using the section-level mean hit count and the section-level mean absolute percentage deviation averaged across repeated measurements. No significant association was observed between hit count and absolute percentage deviation (Spearman r = −0.080, 95% CI: −0.437–0.299, *P* = 0.673; *n* = 30).

Pearson correlation analysis confirmed a strong negative association between true area and percentage deviation for manual planimetry (r ranging from −0.92 to −0.96, *p* < 0.05), meaning that the underestimation grew systematically with section size and geometric complexity (Table 3). For point counting, the correlations were weak and inconsistent; only one round reached statistical significance. Additionally, Spearman correlation showed no significant relationship between absolute percentage deviation and hit count (r = −0.080, *p* = 0.673), indicating that the fixed 1.25 cm grid maintained stable accuracy across the entire range of section sizes (Figure 4).

**Table 3:** Pearson correlations between predefined true area and percentage deviation for point counting and manual planimetry.

Observer		Point counting	Planimetry
Observer 1	E1	−0.17	−0.96*
	E2	−0.30	−0.92*
Observer 2	E1	0.23	−0.95*
	E2	−0.39*	−0.95*

Percentage deviation was calculated as (estimated−true)/true×100. E1, first measurement round; E2, second measurement round.

* *p* < 0.05.

### 3.3 Methodological precision and efficiency

Both techniques were highly repeatable, with intra-observer ICCs above 0.98 (Table 1). A two-way repeated-measures ANOVA showed a significant main effect of method on measurement time [F(1,29) = 511.4, *P* < 0.0001]. For Observer 1, the mean time per section was 94.6 ± 21.8 s for point counting and 195.1 ± 45.9 s for manual planimetry. For Observer 2, the corresponding values were 101.6 ± 18.2 s and 318.4 ± 70.5 s, respectively. Each mean and standard deviation was calculated from 30 section-level values obtained by averaging the two repeated measurements for each section. Thus, point counting required less time than manual planimetry for both observers.

## 4 Discussion

Using a controlled 2D nominal reference model within the Cavalieri framework, this study evaluated the measurement behavior of manual planimetry and point counting—two foundational techniques in dental morphometry (10, 15). Both methods were highly repeatable, but manual planimetry systematically underestimated the true area, with the bias increasing as sections became larger and more fragmented. Point counting exhibited substantially smaller bias and greater efficiency under the specific workflow conditions tested. A critical caveat is that the two methods were implemented on different media: planimetry relied on scanned digital images, whereas point counting was performed directly on physical sections. Consequently, the observed underestimation in planimetry may reflect a composite of tracing error, scanning-induced dimensional distortion, thresholding effects, and calibration inaccuracies, rather than tracing bias alone. We therefore limit our conclusion to the specific implementations tested and do not claim that point counting is fundamentally or intrinsically superior at the pure mathematical level.

The most prominent error pattern was the persistent negative bias of manual planimetry. This likely arises from an “inward-tracking” tendency: to avoid including background pixels, operators trace slightly inside the true boundary (16, 17). While the present 2D physical model does not replicate the grayscale blending, noise, or partial-volume effects of clinical CBCT or micro-CT images, this inward-tracking tendency conceptually parallels operator-dependent behaviors reported in digital segmentation workflows. For instance, Camargo et al. (18) reported that operator-dependent thresholding in CBCT root canal imaging led to systematic area overestimation and perimeter underestimation compared with micro-CT, emphasizing that manual delineation carries inherent bias even in digital workflows. Whether the magnitude and direction of this bias directly translate to clinical imaging, however, remains to be established in studies that explicitly control for scanner-specific factors such as voxel resolution, reconstruction algorithms, and metal artifacts.

The high repeatability of manual planimetry (ICC > 0.98) is consistent with earlier reports, yet it does not guarantee accuracy (7, 19). This precision–accuracy dissociation is a well-known methodological pitfall. If confirmed in clinical datasets, our finding that point counting exhibits lower systematic bias than manual planimetry at the isolated 2D level would support the broader principle that software and segmentation protocol profoundly affect reliability in CBCT-based volumetric analyses. A CBCT volumetric study (20) found that software choice significantly influenced tooth volume measurement accuracy (*P* < 0.001), with 3D Slicer yielding a bias of 4.9% compared with 11.0% for ITK-SNAP, though neither matched planimetry performed by experienced clinicians. By isolating the 2D plane, our results suggest that, under the specific conditions tested, where point counting avoided the digitization step, it achieved smaller bias. However, a same-medium comparison (e.g., virtual point counting on the identical scanned images) would be required to definitively disentangle medium-related from method-related effects. Therefore, the observed differences cannot be attributed solely to the measurement principle itself and should be interpreted within the context of the complete experimental workflow adopted in this study.

This finding aligns with the operational principles of the two methods. Point counting is a discrete binomial process, a point is either a hit or a miss, making it less sensitive to complex geometries (13, 21). Manual planimetry, in contrast, requires continuous tracing and is acutely sensitive to both contour complexity and operator caution. This explains the systematic bias and the marked inter-operator time differences in the planimetry group (7, 19, 22, 23). Although subjectivity is generally attributed to low-contrast clinical images, our data demonstrate that negative bias persists even under ideal, high-contrast conditions (24). The consistency of this result across artificial sections with predefined nominal areas and clinical CT data underscores that manual planimetry and its digital equivalents are prone to systematic error when boundaries are irregular or fragmented.

A key strength of our study is the deliberate exclusion of z-axis confounding factors such as section thickness, partial-volume effects, and interpolation algorithms. Prior comparative work relying on 3D volumetric references often confounds 2D area errors with 3D reconstruction errors (25, 26). In the present implementation, point counting produced lower measurement bias. Recent volumetric studies in oral CBCT support the robustness of stereological approaches. Taşyapan et al. (9) found no significant difference between planimetry and point counting for volume calculation of intraosseous defects under varying section orientations and slice intervals (*P* = 0.628), with high ICCs for orientation (0.951) and slice interval (0.923), and all volumes were consistent with Archimedes' principle. However, their study operated at the volumetric level, where z-axis sampling factors (section thickness, slice interval, and 3D reconstruction) can mask underlying 2D area-estimation errors. Our isolated 2D design deliberately removes these confounders, thereby revealing the observed section-level bias that volume-level analyses may have obscured. Thus, the two findings are complementary rather than contradictory: once z-axis sampling factors are controlled at the volume level, both methods can achieve acceptable volumetric accuracy, whereas differences at the fundamental 2D level emerge primarily in efficiency and section-level bias profiles.

Automated segmentation in dental imaging is advancing rapidly. Ntovas et al. (27) demonstrated that AI-driven tooth segmentation on CBCT images achieves accuracy comparable to manual segmentation by experienced clinicians while significantly improving time efficiency (*P* < 0.05), although manual segmentation still resulted in less RMS deviation compared to AI methods. Similarly, a systematic review (28) reported pooled Dice similarity coefficients of 0.94 for mandible segmentation, 0.925 for teeth segmentation, and 0.907 for maxilla segmentation from CBCT and CT scans, confirming that deep-learning models achieve high accuracy in segmenting dental and maxillofacial structures, comparable to expert manual segmentation. However, the present findings emphasize an important nuance: even the most accurate automated segmentation must rely on a ground-truth reference for validation. Point counting, by providing a grid-based 2D estimate under the tested conditions, may serve as a useful reference for training and validating AI segmentation models, particularly for fragmented or irregular anatomical targets where manual planimetry's systematic bias would be most detrimental. Mandić et al. reinforced this perspective, noting that AI-based segmentation has become the state-of-the-art in many applications, yet the need for robust validation against unbiased references remains paramount (22).

The time efficiency advantage of point counting observed in this study is substantial (approximately threefold faster per section for Observer 2). Although this advantage was demonstrated under controlled experimental conditions, it raises the possibility that similar efficiency gains might be realized in clinical or histomorphometric workflows once the method is validated on real tissue sections or imaging datasets. Duan et al. (29) developed a deep learning auto-segmentation-assisted contour QA system that reduced manual review time by 75%, demonstrating that hybrid workflows combining automated methods with unbiased validation can achieve both accuracy and efficiency. In oral morphometry specifically, Melih Ozdede et al. (20) reported that CBCT device type, voxel size, and segmentation software all influence tooth volume measurement accuracy, with the optimal combination (Planmeca device + 0.1-mm voxel + 3D Slicer) showing no statistical difference from water displacement gold standard (*p* = 0.467). The time-efficient nature of point counting makes it particularly suitable for high-throughput morphometric studies and for training datasets where large numbers of sections require annotation.

Looking forward, the integration of point counting principles into automated segmentation algorithms may represent an important methodological synergy. A deep CNN-based automated maxillofacial segmentation model has been reported to reduce segmentation time from 132.7 min manually to 39.1 s automatically, with a Dice similarity coefficient of 92.6% (30). However, these AI-related implications remain indirect in the present study because no automated segmentation method was tested. Incorporating point counting-based area estimates as validation targets could help assess whether automated segmentations inherit the systematic underestimation bias characteristic of manual planimetry, although this would require validation across multiple realizations and imaging conditions. Moreover, the strong negative correlation between section size and percentage deviation in planimetry (r ranging from −0.92 to −0.96, *P* < 0.05) suggests that bias is both systematic and predictable, raising the possibility of developing correction factors or calibration protocols for planimetry-based workflows where point counting cannot be directly applied (16).

These findings must be interpreted considering the model's simulated nature. The predefined areas were nominal geometric reference values rather than substrate-independently measured physical ground-truth areas. While isolating geometric error, this physical model does not replicate the gray-scale blending, noise, or soft-tissue contrast ambiguities of clinical MRI or CBCT data. Furthermore, CBCT-specific factors, such as scanner type, voxel resolution (0.1-mm vs. 0.2-mm), reconstruction algorithms, and the presence of metal artifacts from implants, fillings, or orthodontic brackets, can introduce additional layers of measurement error not captured in our controlled 2D model (6, 8). While this controlled model effectively isolated geometric measurement error, the findings derive from a single paradigm with standardized contrast and defined borders. Future validation in diverse clinical datasets, encompassing varied anatomical geometries, tissue contrasts, and imaging modalities, is needed to confirm generalizability. Extending the comparison to 3D volumetric reconstructions with varied slice parameters would also clarify how 2D section-level biases propagate into Cavalieri-based estimates. Nonetheless, for fragmented or irregular targets in this controlled 2D paradigm, point counting outperformed manual planimetry in both efficiency and systematic bias reduction. Caution is warranted, however, as these results emanate from a single high-contrast model and await replication across varied clinical and histological contexts (3, 10).

## 5 Conclusions

Using a predefined 2D nominal reference model, this study compared the accuracy, precision, and efficiency of manual planimetry and point counting. We acknowledge that the two methods were implemented on different media: planimetry on scanned digital images and point counting directly on physical sections. Consequently, the observed differences reflect the performance of these specific implementations rather than an intrinsic superiority of one method over the other.

Under the controlled conditions tested, point counting demonstrated smaller bias and higher efficiency, whereas manual planimetry, although highly repeatable, systematically underestimated area as section size and fragmentation increased. These results indicate that grid-based point counting may provide a useful and efficient reference approach for morphometric assessment under controlled, high-contrast conditions. Caution is warranted when extrapolating these findings to clinical imaging, where grayscale blending, noise, and soft-tissue contrast ambiguities may alter measurement behavior. These conclusions are drawn from a single model realization and should be confirmed by independent replication before broad generalization.

## Data Availability

The original contributions presented in the study are included in the article/Supplementary Material, further inquiries can be directed to the corresponding authors.

## Supplementary material

SUPPLEMENTARY TABLE S1 Raw section-level measurement and timing data for point counting and manual planimetry across two observers and two repeated rounds.
